# Per-Ingvar Brånemark (1929–2014): A Homage to the Father of Osseointegration and Modern Dentistry

**DOI:** 10.7759/cureus.68655

**Published:** 2024-09-04

**Authors:** Swati Sharma, Sarat Ravi Kiran, Pankaj Kumar, Rama Shankar, Amitabh Kumar Upadhyay

**Affiliations:** 1 Dentistry, Tata Main Hospital, Jamshedpur, IND; 2 Oral and Maxillofacial Surgery, Tata Main Hospital, Jamshedpur, IND; 3 Endodontics, Tata Main Hospital, Jamshedpur, IND; 4 Medical Oncology, Tata Main Hospital, Jamshedpur, IND

**Keywords:** osseointegration, titanium, modern dentistry, branemark, "historical vignette"

## Abstract

Dr. Professor Per-Ingvar Brånemark (Branemark), a Swedish professor of anatomy, is widely acknowledged as a pioneer in modern implant dentistry. His accidental discovery of the strong bond between titanium and bone, which he named "osseointegration," signifies a revolutionary progression in dentistry. This significant contribution has earned him global recognition among scholars and the general public. His work, which spans across disciplines, has introduced a new era of implant reconstruction and fostered the formulation of principles rooted in bone biology. This interdisciplinary advancement has paved the way for diverse craniofacial and orthopedic applications, including facial prostheses and limb replacements, owing to titanium's biocompatibility within the human body. This article stands as a tribute to Brånemark and his remarkable innovation. Despite not being a dentist, Brånemark, an orthopedic surgeon, has left an enduring legacy in dental implantology that continues to impact the field today and will undoubtedly do so in the future, deserving lasting recognition.

## Introduction and background

Dr. Professor Per-Ingvar Brånemark (Branemark) was born on May 3, 1929, in Gothenburg, Sweden. He was a Swedish physician and researcher widely recognized as the "father of modern dental implantology [1]." Brånemark, a distinguished Swedish professor of anatomy, serendipitously discovered a strong connection between titanium and bone, which he termed "osseointegration [2-5]." His groundbreaking discovery and significant contributions to the field have earned him international acclaim among scholars and individuals alike. On December 20, 2014, in Gothenburg, Sweden, the renowned individual died unexpectedly when he was 85 due to a heart attack [1,6-8]. Here, we pay homage to the great scientist who has contributed immensely to the healthcare industry.

## Review

Scientific contributions

In the course of orthopedic experiments on rabbit legs, Brånemark made a significant breakthrough. He observed that titanium has a high propensity to integrate with bone, a phenomenon he termed osseointegration [2]. This finding might have been disregarded by many investigators and has since proven to be significant. His comprehensive approach to examining all variables in his research enabled him to observe the formation and escape of multipotent undifferentiated cells from the bone marrow upon exposure to implantable titanium chambers. His research was based on an intimate understanding of the inflammatory process and bone healing. Brånemark's research revealed that pure titanium is biocompatible, as it does not elicit significant inflammatory or adverse reactions in skin or bone tissues. This finding is crucial for establishing long-lasting bonds between superficial prostheses and for craniofacial restoration.

What is Osseointegration

Osseointegration refers to the process by which non-vital titanium components are structurally integrated with living bone. To achieve a prosthesis's skeletal anchorage, it is imperative to prevent relative movements between the implant and anchoring tissue. Moreover, properly loading the anchoring bone is essential to facilitate adequate remodeling and provide a stable anchorage platform [9]. This research is underpinned by examining bone and marrow tissue vascular systems alongside an assessment of the impact of surgical techniques on the regenerative and remodeling capacity of bone tissue following trauma and functional load. The efficacy of osseointegration in preclinical and clinical trials has facilitated its adaptation for diverse orthopedic applications, such as facial prostheses (ear, eye, and nose) and limb replacements for amputees [9].

Professional journey and achievements

In 1956, Professor Brånemark received his medical degree from Lund University Sweden, and subsequently, in 1959, he obtained his PhD. In 1952, while conducting orthopedic experiments on rabbit legs, Brånemark unintentionally serendipitously discovered the concept of osseointegration. In subsequent years, he took on the position of Anatomy Professor at Gothenburg University, concurrently acting as the Director of the Experimental Biology Laboratory from 1963 to 1977. During this timeframe, Brånemark, in partnership with a multidisciplinary team comprising medical biologists, technologists, and engineers, embarked on the conceptualization and realization of oral implants intended for the reconstruction of edentulous jaws. The treatment of the first utterly edentulous patient in 1965 marked a significant milestone, ushering in a modern age in dental implant rehabilitation and spreading the foundation of advancing implant acceptance principles rooted in bone biology [1,6-8].

Brånemark founded the Institute of Applied Biotechnology in Gothenburg in 1977, which served as the central hub for pioneering research in bone biology, implant design, and clinical treatment. Initially, Brånemark's research on osseointegration faced skepticism, as prevailing medical knowledge implied that the human body was generally hostile to foreign materials, often resulting in inflammation. Consequently, his grant applications for studies on bone-anchored implants encountered frequent rejection. Brånemark persevered and eventually secured Health Funding from the United States National Institutes. Consequently, implants by Branemark have formed the foundation of dental implantology since their pioneering extension of osseointegration into dentistry during the 1970s. The introduction of machined titanium implants at the 1982 Toronto Osseointegration Conference marked a milestone, enabling their broader application in clinical settings. Subsequently, "osseointegration" was formally introduced and internationally recognized [1,6-8].

On December 19, 2003, Brånemark was honored with the Doctor of Dental Science degree. The Chancellor, the Honorable Justice Kim Santow, conferred the degree upon Brånemark. This honor was given by the resolution made by the Senate of the University's meeting on April 8, 2002, where it was resolved that Brånemark be awarded the degree of Doctor of Dental Science (honoris causa) [1,6-8].

Osseointegration is closely associated with Brånemark and forms the biological foundation for reliable implant treatment. The Swedish government acknowledged the treatment of complete edentulism as a significant advancement in healthcare. It supported this health need, making Sweden the only country to eliminate edentulism effectively. The work of Brånemark created widespread international interest, leading to the initiation of treatment teams in Toronto in 1980 and Sydney and Perth in 1981. Following this, other centers also began offering this treatment, and in 1988 in Leuven, the European Osseointegration Training Centre was established [1,6-8].

The Brånemark Osseointegration Centre

The Brånemark Osseointegration Centre was established in 1989 in Gothenburg, Sweden, as a specialized clinic focusing on reconstructive surgery and tissue-integrated prostheses. The clinic has been a leader in making oral implant restoration a standard procedure in clinical practice. Following the success of oral implant reconstruction, the clinic expanded its work to orthopedics for small and large joint replacement. It started with small joints, like the hand's interphalangeal joints, for progressive osteoarthritic disease, then moved on to replacing diseased knees, elbows, and hips. The clinic employed osseointegration techniques to assist individuals with limb amputations. This innovative approach involved the utilization of a bone-anchored prosthesis, which not only restored functionality but also facilitated a previously unattainable "sense of function," now referred to as osseoperception. This advancement significantly enhanced the efficacy of prosthetic rehabilitation. The oral implantology principles have been successfully utilized in maxillofacial rehabilitation and bone-supported hearing aids. Patients have experienced improved confidence in prosthesis use and social interactions. Additionally, the advanced surgical technique has effectively managed complex oro-facial irregularities using implants [1,6-8].

In 1992, he received the prestigious "Swedish Society of Medicine's Soederberg Prize," often referred to as the "mini-Nobel," and was also awarded the esteemed medal for technical innovation from the Swedish Engineering Academy. His work in dental implants was recognized by the Harvard School of Dental Medicine with a dedicated medal. Additionally, he held more than 30 honorary recognitions in Europe and North America, which included the Honorary Fellowship of the Royal Society of Medicine in the United Kingdom [1,6-8].

Improvements in implants

Brånemark consistently prioritized patient welfare, conducting post-surgical clinical assessments to ensure patients received appropriate care, irrespective of the surgery location. He maintained long-term communication with his patients following treatment. Osseointegrated implants have significantly advanced dentistry, offering reliable care for completely and partially edentulous patients. Before the development of osseointegration, implant dentistry faced challenges in achieving predictable results. Dr. Brånemark, recognizing the limits of his expertise, actively engaged accomplished researchers, business advisors, and clinicians to avail the best counsel [1,6-8].

Why did Brånemark never receive the Nobel Prize?

In 2006, a member of a Belgian university was invited to support Brånemark for the Nobel Prize, a testament to the global collaboration in dental research. Michele Aerden, the president of the FDI World Dental Federation from Belgium, also strongly endorsed this nomination. Regrettably, Brånemark expired in December 2014, and the Nobel Prize cannot be given after a person's death, a loss felt by the entire dental research community [8]. Notably, while convincing, the Brånemark research team was not the first to acknowledge titanium as a conceivable material for implantation. In the years prior to World War II, a pioneering proposition was put forth by a British research team [10], with subsequent support from a post-war U.S. researcher [11]. These early researchers observed a "tendency for the bone to fuse with titanium," a foresight we now appreciate as a critical milestone in dental implant research [11].

Pioneering researchers in dentistry and medicine, such as Beder and Ploger [12] and Emnéus and Stenram [13], demonstrated bone's favorable tolerance to implanted titanium pieces. This finding was substantiated by a comprehensive elucidation of the intersurface phenomena between titanium and bone under various experimental states in a 1969 publication by Brånemark and his team [14]. This comprehensive understanding of the interface phenomena informs the scientific basis of dental implants.

The seminal research conducted by Brånemark and his team has significantly influenced medical innovation. Their pioneering work was initially presented in a seminal publication in 1977 [4] and further expounded upon in another pivotal publication in 1981 [15]. This research has been comprehensively documented in "The Osseointegration Book," published in 2005 [16]. After transferring the manufacturing rights to a Swedish company, Professor Brånemark engaged in commercial pursuits and secured patents in Sweden and the United States in 1979 [1]. This occurred shortly after Straumann submitted a patent for a titanium-based dental implant in the United States, despite numerous prior filings for titanium and titanium alloy dental implants. He believed in the possibility of achieving anything and has left an indelible mark on his profession. Brånemark sincerely appreciated English literature, classical music, and theater. Despite his dedication to his work, he never made time for vacations, as noted by his wife, Barbro [1]. Widely regarded as a genius, he not only established a global implant market valued at more than 30 billion dollars but also did so through rigorous research, comprehensive data analysis, a profound grasp of biology and mechanics, and practical clinical application. It is essential to acknowledge that although Brånemark's name has become linked with a specific brand of dental implants, his contributions extend beyond his association with a particular company.

## Conclusions

Brånemark, despite not being a dentist, commands immense respect from researchers across various biomedical fields, particularly in dentistry, owing to his seminal contributions to investigating the efficacy of implants. Brånemark, with his charm, brilliance, and boundless imagination, has transformed the field of dentistry. He was committed to finding tailored solutions for those in need, prioritizing their well-being over financial considerations. His pioneering research has been effectively translated into clinical trials, leading to significant positive outcomes for countless individuals with dental issues. Most knowledgeable dentists routinely recommend osseointegrated implants to their patients as part of their daily practice. The titanium implants, a testament to his exemplary research endeavors, augmented his study of osseointegration and have significantly impacted dentistry as well as other branches of medicine.

## References

[1] Kim TI (2014). A tribute to Dr. Per-Ingvar Brånemark. J Periodontal Implant Sci.

[2] Brånemark PI (1961). Experimental investigation of microcirculation in bone marrow. Angiology.

[3] Branemark PI, Zarb GA, Albrektsson T, Rosen H (1986). Tissue-integrated prostheses. Osseointegration in clinical dentistry. Plast Reconst Surg.

[4] Brånemark PI, Hansson BO, Adell R, Breine U, Lindström J, Hallén O, Ohman A (1977). Osseointegrated implants in the treatment of the edentulous jaw. Experience from a 10-year period. Scand J Plast Reconstr Surg Suppl.

[5] Brånemark PI (1983). Osseointegration and its experimental background. J Prosthet Dent.

[6] Ali Ali (2019). History of dental implants, in memoriam: Dr. Per-Ingvar Branemark, the man who made people smile. Int J Adv Res Ideas Innov Technol.

[7] Nevins M, Janson T (2015). Reminiscences of the late professor Per-Ingvar Brånemark. J Periodontol.

[8] Jokstad A (2017). Why did professor Per-Ingvar Brånemark never receive the nobel prize in medicine?. Clin Exp Dent Res.

[9] Jayesh RS, Dhinakarsamy V (2015). Osseointegration. J Pharm Bioallied Sci.

[10] Bothe RT, Beaton LE, Davenport HA (1940). Reaction of bone to multiple metallic implants. Surg Gynecol Obstet.

[11] Leventhal GS (1951). Titanium, a metal for surgery. J Bone Jt Surg.

[12] Beder OR, Ploger WJ (1959). Intraoral titanium implants. Oral Surg Oral Med Oral Pathol.

[13] EM H, ST U (1960). Reaction of tissues to alloys used in osteosynthesis. Experimental, histological examination of reaction of soft tissues in animals. Acta Orthop Scand.

[14] Brånemark PI, Adell R, Breine U, Hansson BO, Lindström J, Ohlsson A (1969). Intra-osseous anchorage of dental prostheses. I. Experimental studies. Scand J Plast Reconstr Surg.

[15] Albrektsson T, Brånemark PI, Hansson HA, Lindström J (1981). Osseointegrated titanium implants. Requirements for ensuring a long-lasting, direct bone-to-implant anchorage in man. Acta Orthop Scand.

[16] Brånemark PI The biologic origin of osseointegration. The osseointegration book: from calvarium to calcaneus.

